# A quantitative method to monitor STING degradation with dual-luciferase reporters

**DOI:** 10.1247/csf.25011

**Published:** 2025-04-19

**Authors:** Tsumugi Shoji, Kanako Sato, Ayumi Shinojima, Shogo Koide, Ruri Shindo, Kazune Hongo, Kojiro Mukai, Yoshihiko Kuchitsu, Tomohiko Taguchi

**Affiliations:** 1 Laboratory of Organelle Pathophysiology, Department of Integrative Life Sciences, Graduate School of Life Sciences, Tohoku University, Sendai, Japan

**Keywords:** innate immunity, STING, membrane traffic, lysosomal degradation, luciferase

## Abstract

Stimulator of interferon genes (STING) triggers the type I interferon and inflammatory responses against a variety of DNA pathogens, which is essential to limiting viral infection and replication. STING activates the downstream kinase TBK1 at the trans-Golgi network (TGN) and is degraded at lysosomes through a process called lysosomal microautophagy. Impaired STING targeting to lysosomes results in the prolonged inflammatory signal, which may be associated with a variety of neurodegenerative and autoinflammatory diseases. Thus, development of methods to quantify STING degradation helps understand the mechanism of lysosomal microautophagy and its related diseases. Here we report a quantitative method to monitor STING degradation with two luciferases, firefly luciferase (FLuc) and Nanoluciferase (NLuc). The expression plasmid is composed of FLuc, a P2A self-cleavage site, and NLuc-tagged STING. FLuc intensity reflects the total amount of translated protein, serving as an internal control, while NLuc intensity corresponds to the amount of STING. Comparison of the NLuc/FLuc ratios at different time points after STING stimulation revealed the kinetics of decay of STING levels in live cells. This method should provide a useful complement to western blotting and fluorescence-activated cell sorter (FACS) analysis presently used to monitor STING degradation.

## Graphical Abstract

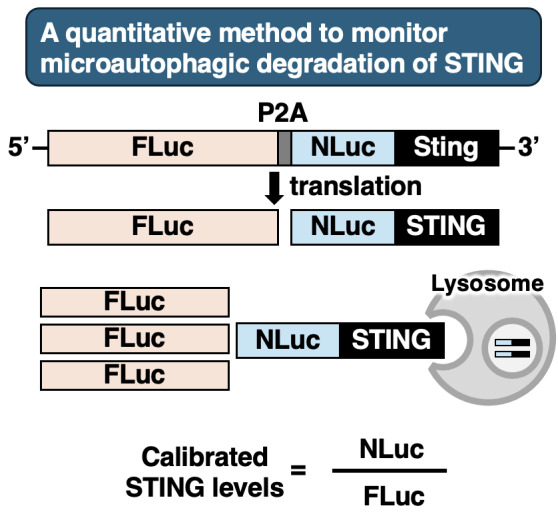

## Introduction

The detection of microbial pathogens with nucleic acid sensors is one of the central strategies of innate immunity (Palm and Medzhitov, 2009; Takeuchi and Akira, 2010). Cyclic GMP-AMP synthase (cGAS) is a sensor for double-stranded DNA (dsDNA) in the cytosol (Sun *et al.*, 2013). It synthesizes cyclic GMP-AMP (cGAMP) with ATP and GTP (Wu *et al.*, 2013), which induces the type I interferons and proinflammatory cytokines through the cGAMP sensor STING, an ER-localized transmembrane protein (Ishikawa *et al.*, 2009; Ishikawa and Barber, 2008) [also known as MITA (Zhong *et al.*, 2008), ERIS (Sun *et al.*, 2009), MPYS (Jin *et al.*, 2008), or TMEM173]. After STING binds to cGAMP, STING translocates to the Golgi and undergoes palmitoylation at Cys88 and Cys91 (Hansen *et al.*, 2018; Matsumoto *et al.*, 2023; Mukai *et al.*, 2016). Palmitoylated STING forms clusters in cholesterol- and sphingomyelin-rich lipid microdomains in the TGN, promoting TBK1 autophosphorylation and activation on STING (Kemmoku *et al.*, 2022, 2024; Takahashi *et al.*, 2021). The activated TBK1 then phosphorylates the transcription factor interferon regulatory factor 3 (IRF3) (Tanaka and Chen, 2012). Phosphorylated IRF3 by TBK1 dimerizes and translocates into the nucleus to induce transcription of genes that encode type I interferons such as interferon-β (IFN-β). STING also induces proinflammatory response via NF-κB by the activation of TBK1 and IKKε (Balka *et al.*, 2020; Fischer *et al.*, 2025).

After exit from the TGN, STING moves to recycling endosomes (REs) where STING undergoes K63-linked polyubiquitination at Lys288 (Kuchitsu *et al.*, 2023). This ubiquitination is required for the endosomal sorting complexes required for transport (ESCRT)-driven lysosomal microautophagic degradation of STING (Kuchitsu *et al.*, 2023; Kuchitsu and Taguchi, 2024). The impaired post-Golgi membrane traffic of activated STING or dysfunction of the ESCRT complex results in the prolonged inflammatory signals, which may be associated with a variety of autoinflammatory and neurodegenerative diseases including amyotrophic lateral sclerosis (ALS) (McCauley *et al.*, 2020) and hereditary spastic paraplegia (HPS) (Farazi Fard *et al.*, 2019; Gu *et al.*, 2020; Lin *et al.*, 2019; Zivony-Elboum *et al.*, 2012). Thus, STING needs to be degraded in a timely manner. Indeed, in various cell types, STING degradation occurs within a time window of several hours after STING activation. The molecular mechanism that regulates STING degradation has just begun to be elucidated.

Routinely, STING degradation is evaluated by western blotting or FACS analysis. Both methods have some disadvantages. Because they are not high-throughput methods, genome-wide siRNA screening or chemical library screening cannot be performed. Another disadvantage is related to the post-translational modifications of STING. As STING is subjected to K63-linked polyubiquitination at REs (Kuchitsu *et al.*, 2023), the band of STING becomes upshifted and smeared in western blotting. Therefore, the amount of K63-linked polyubiquitinated STING, which is the substrate for lysosomal degradation, cannot be accurately quantified by western blotting.

In the present study, we report a quantitative method to monitor STING degradation with two luciferases, FLuc and NLuc. The method is not affected, in principle, with post-translational modifications of STING and can be performed on a 96-well/384-well plate, enabling high-throughput screening. Thus, the method should provide a useful complement to the western blotting and FACS analysis presently used to monitor STING degradation.

## Results

### Development of a quantitative assay to monitor STING degradation with dual luciferases

**Fig. 1:**
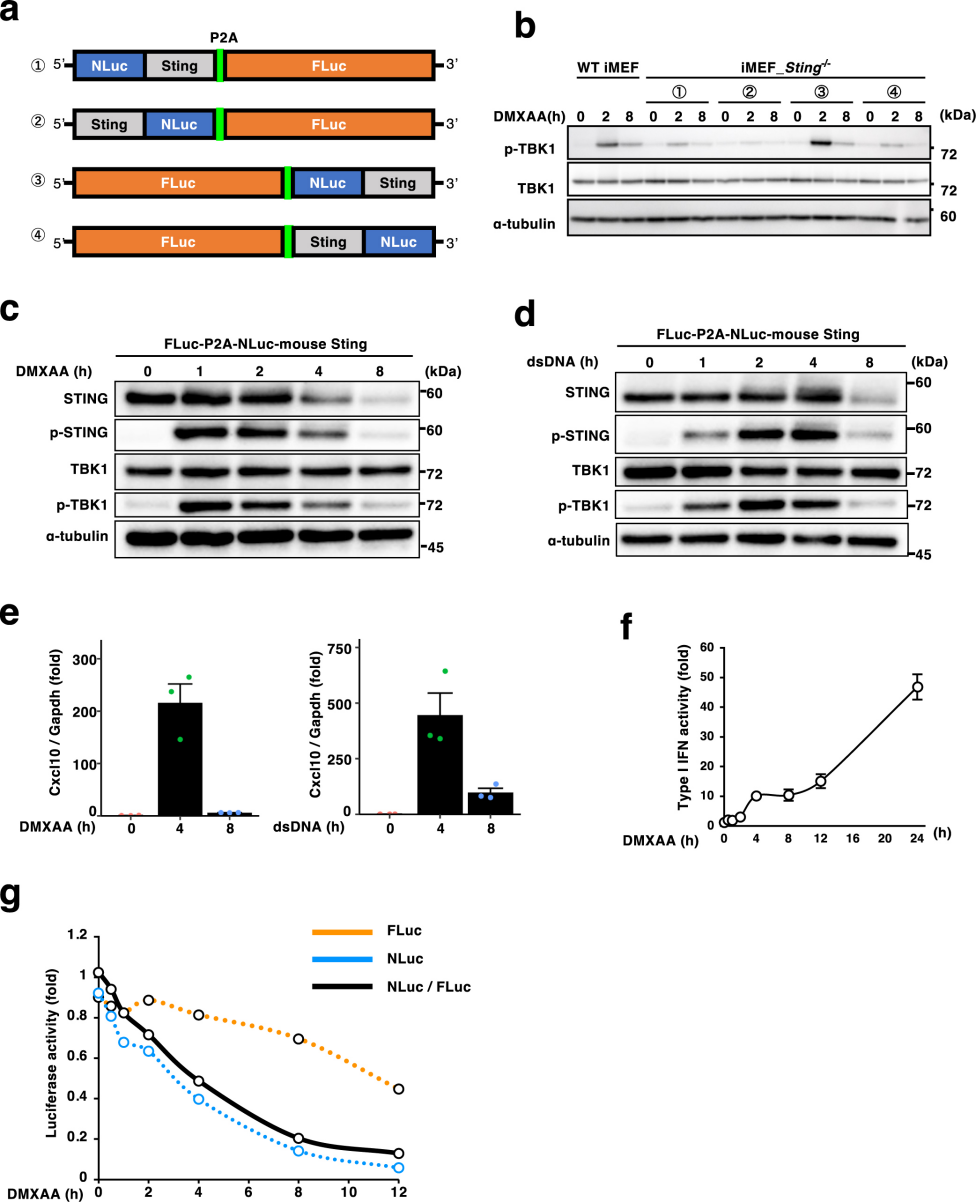
a, Schematic illustration of the plasmids encoding NLuc-mouse Sting-P2A-FLuc, mouse Sting-NLuc-P2A-FLuc, FLuc-P2A-NLuc-mouse Sting, and FLuc-P2A-mouse Sting-NLuc. b, Cells expressing the individual construct shown in (a) were stimulated with DMXAA for the indicated times. Cell lysates were analyzed by western blot. c–g, *Sting*^–/–^ iMEFs stably expressing FLuc-P2A-NLuc-STING were stimulated with double-stranded DNA or DMXAA for the indicated times. Cell lysates were analyzed by western blot (c, d). The expression of Cxcl10 was quantified with qRT-PCR in (e). The activity of the type I interferon (IFN) in the cell supernatants in (f). The luminescence of FLuc and NLuc was quantified at each time point, and the NLuc/FLuc ratio was plotted in (g).

To quantify STING degradation, we utilized the Nano-Glo^®^ Dual-Luciferase^®^ Reporter Assay. Several STING expression plasmids were designed, each encoding FLuc, NLuc-tagged mouse STING, and a P2A self-cleavage site in a different order (Fig. 1a). We reconstituted immortalized *Sting*^–/–^ mouse embryonic fibroblasts (iMEFs) with these plasmids and examined if NLuc-tagged STING, like endogenous STING, was activated properly and degraded in a time window within several hours (Gui *et al.*, 2019; Konno *et al.*, 2013; Kuchitsu *et al.*, 2023). The cells were stimulated for several hours with DMXAA, a membrane-permeable rodent-specific STING agonist, and the cell lysates were examined for emergence of phosphorylated TBK1 (p-TBK1), the indication of STING activation. Among the four expression plasmids tested, only FLuc-P2A-NLuc-STING was found to activate TBK1 2 hours after stimulation (Fig. 1b). The band corresponding to NLuc-STING mostly disappeared 8 hours after stimulation either with DMXAA (Fig. 1c) or dsDNA (Fig. 1d). NLuc-STING induced transcription of Cxcl10 (Fig. 1e) and production of type I interferons (Fig. 1f), showing that NLuc-STING was functional.

We then quantified the degree of STING degradation by measuring activities of FLuc and NLuc in *Sting*^–/–^ iMEFs expressing FLuc-P2A-NLuc-STING. FLuc intensity reflects the total amount of translated protein, serving as an internal control, while NLuc intensity corresponds to the amount of STING. The NLuc/FLuc ratio was calculated at each time point after STING stimulation, and normalized to the ratio obtained from unstimulated cells. As shown (Fig. 1g), the normalized NLuc-STING/FLuc ratio decreased gradually and reduced by 80% 8 hours after DMXAA stimulation.

### Validation of NLuc-STING by fluorescence microscopy

**Fig. 2:**
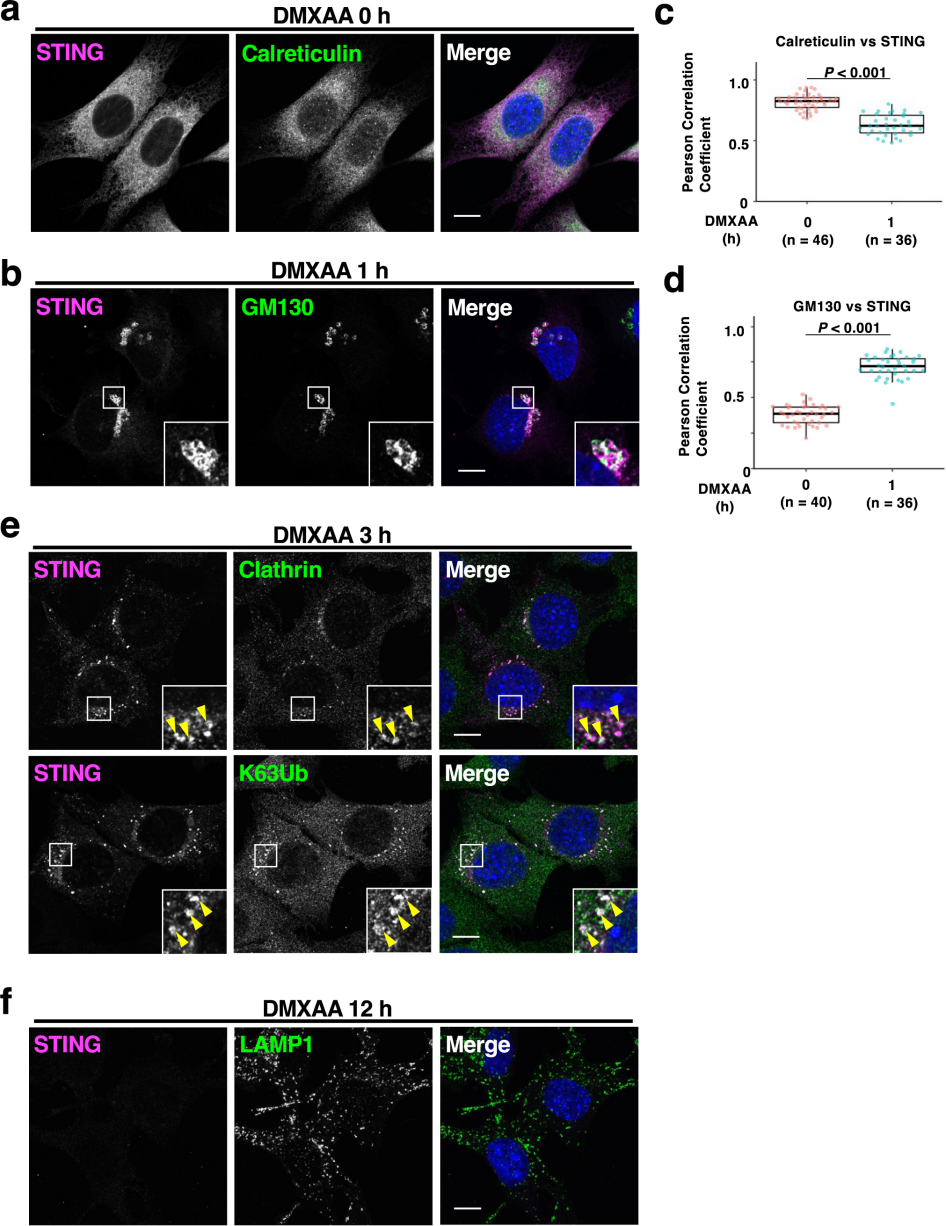
a–f, *Sting*^–/–^ iMEFs stably expressing FLuc-P2A-NLuc-mSting were treated with DMXAA for the indicated times. Cells were immunostained with anti-LgBiT (anti-NLuc), anti-STING, anti-calreticulin in (a), anti-GM130 in (b), anti-clathrin heavy chain in (e), anti-K63 ubiquitin in (e), or anti-LAMP1 antibody in (f), respectively. The Pearson correlation coefficient between STING and calreticulin or between STING and GM130 is shown in (c) or (d).

We validated the subcellular localization of NLuc-STING encoded in FLuc-P2A-NLuc-STING by fluorescence microscopy. After ligand binding, STING translocates from the ER to the Golgi, then to REs (Ishikawa *et al.*, 2009; Kuchitsu *et al.*, 2023; Mukai *et al.*, 2016; Saitoh *et al.*, 2009). At REs, STING undergoes K63-linked polyubiquitination (Fischer *et al.*, 2025; Kuchitsu *et al.*, 2023) and is loaded to clathrin-coated vesicles (Kuchitsu *et al.*, 2023; Liu *et al.*, 2022), which are the substrate for lysosomal microautophagy (Kuchitsu *et al.*, 2023). In unstimulated cells, NLuc-STING colocalized with calreticulin , an ER-localized protein (Fig. 2a). One hour after DMXAA stimulation, NLuc-STING lost the reticular localization and translocated to the perinuclear compartments in which STING co-localized with GM130, a Golgi-localized protein (Fig. 2b). Consistently, the Pearson correlation coefficient of STING with calreticulin significantly decreased, whereas that with GM130 significantly increased after stimulation (Fig. 2c–d). Three hours after DMXAA stimulation, STING localized at punctate structure at which STING colocalized with clathrin heavy chain and K63-linked ubiquitin (Fig. 2e), suggesting that STING localized at REs and/or clathrin-coated vesicles. Twelve hours after stimulation, the signal of NLuc-STING mostly diminished, suggesting its degradation (Fig. 2f).

In sum, NLuc-STING behaved like endogenous STING with respect to its subcellular trafficking route after stimulation.

### Validation of NLuc-STING by knockdown of genes essential for STING translocation or degradation

**Fig. 3:**
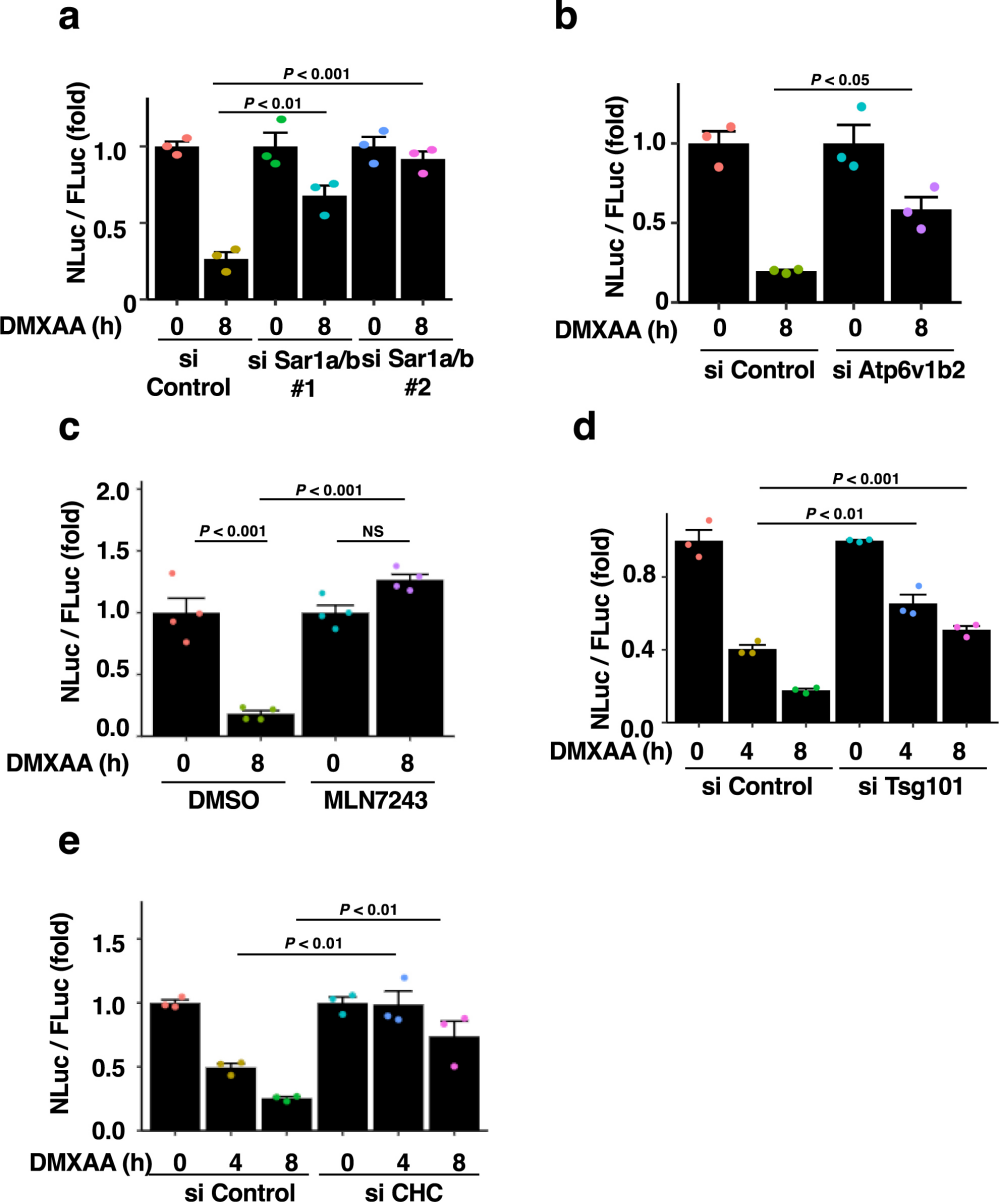
a–b, *Sting*^–/–^ iMEFs stably expressing FLuc-P2A-NLuc-mSting were treated with the indicated siRNAs for 64 h, and then stimulated with DMXAA for 8 h. The luminescence of FLuc and NLuc was quantified, and the NLuc/FLuc ratio was plotted. Data are presented as mean ± standard error of the mean. c, *Sting*^–/–^ iMEFs stably expressing Fluc-P2A-NLuc-STING were treated with or without MLN7243 (2 μM) for 30 min, and then stimulated with DMXAA for 8 h. The luminescence of FLuc and NLuc was quantified, and then Nluc/Fluc ratio was plotted. d–e, *Sting*^–/–^ iMEFs stably expressing FLuc-P2A-NLuc-mSting were treated with the indicated siRNAs for 72 h, and then stimulated with DMXAA for the indicated times. The luminescence of FLuc and NLuc was quantified, and the NLuc/FLuc ratio was plotted. Data are presented as mean ± standard error of the mean.

We further validated NLuc-STING by knockdown of several genes that regulate STING translocation or degradation. Knockdown of Sar1A and Sar1B, which are required for the ER-to-Golgi trafficking (Nakano and Muramatsu, 1989; Shen *et al.*, 1993), significantly suppressed the stimulation-dependent degradation of NLuc-STING (Fig. 3a). The result was consistent with the notion that STING translocation to REs was required for STING degradation (Kuchitsu *et al.*, 2023). Knockdown of Atp6v1b2, a gene encoding a subunit of vacuolar ATPase, suppressed the stimulation-dependent degradation of NLuc-STING (Fig. 3b), suggesting that NLuc-STING, like endogenous STING, was degraded in lysosomes.

STING at REs is subjected to K63-linked polyubiquitination, and loaded into clathrin-coated vesicles. K63-linked polyubiquitinated STING was recognized by TSG101, a subunit of the ESCRT-I complex, and the recognition was essential for STING degradation by lysosomal microautophagy (Kuchitsu *et al.*, 2023). In line with these notions, the treatment of cells with MLN7243 (an inhibitor of ubiquitin-activating enzyme 1) (Balka *et al.*, 2023; Gentili *et al.*, 2023), knockdown of Tsg101, or knockdown of clathrin heavy chain (CHC), suppressed the stimulation-dependent degradation of NLuc-STING (Fig. 3c–e). Collectively, these results endorsed the dual luciferase system with NLuc-STING for the quantitative measurement of STING degradation.

### Development of a quantitative assay to monitor human STING degradation

**Fig. 4:**
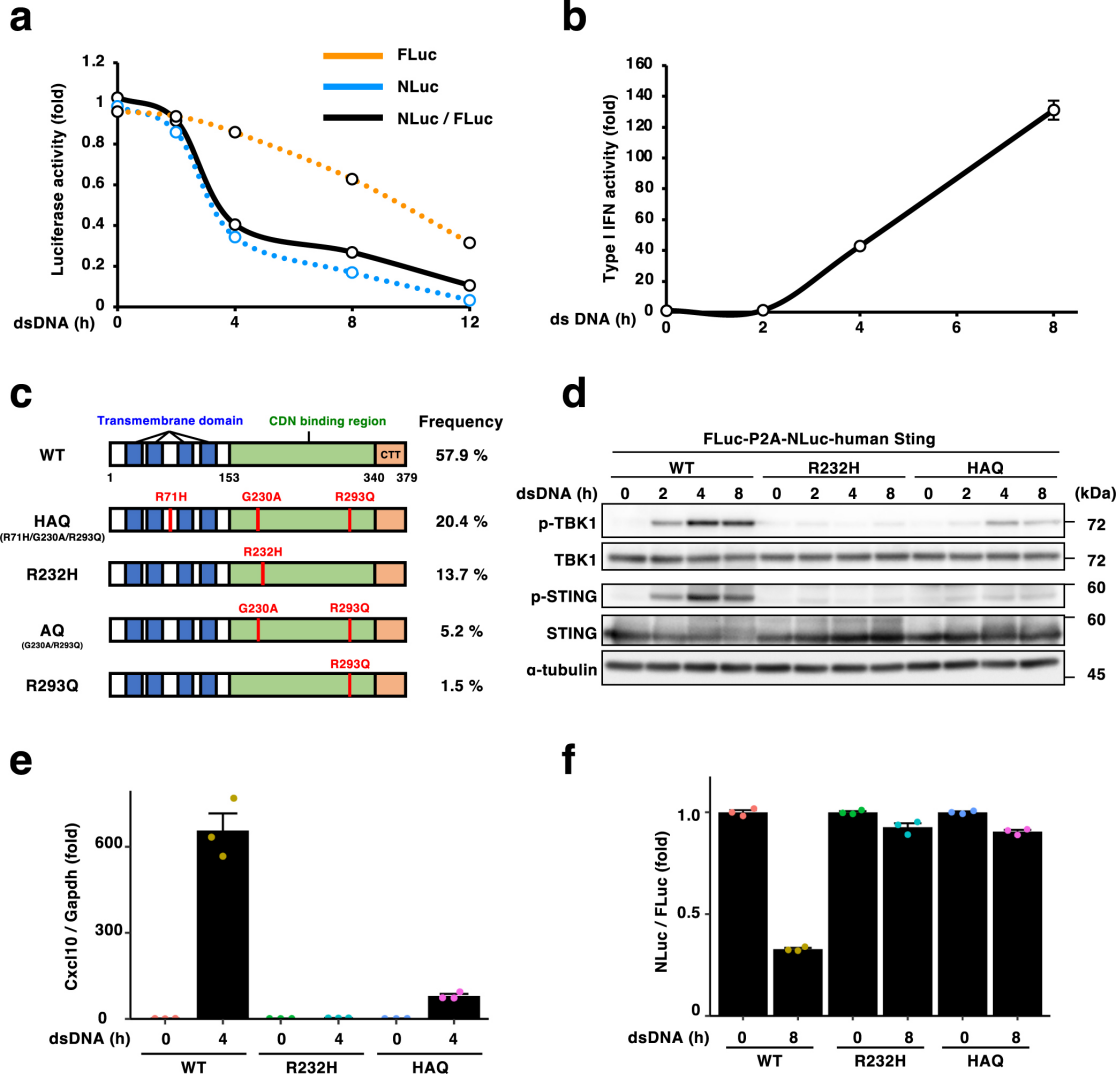
a, *Sting*^–/–^ iMEFs stably expressing FLuc-P2A-NLuc-human Sting were stimulated with double-stranded DNA for the indicated times. The luminescence of FLuc and NLuc was quantified at each time point, and the NLuc/FLuc ratio was plotted. b, Cell supernatants in (a) were analyzed for the activity of type I interferon (IFN). c, Schematic illustration of domain structures of human STING. The allele frequencies are shown on the right. Red lines indicate the positions of amino acid variations. d–f, *Sting*^–/–^ iMEFs stably expressing FLuc-P2A-NLuc-human Sting were stimulated with double-stranded DNA for the indicated times. The cell lysates were analyzed by western blot in (d). The expression of Cxcl10 was quantified with qRT-PCR in (e). The luminescence of FLuc and NLuc was quantified at each time point, and the NLuc/FLuc ratio was plotted in (f).

We sought to extend our experience with mouse STING to human STING. Thus, a expression plasmid encoding FLuc-P2A-NLuc-human STING was designed and the plasmid was expressed in immortalized *Sting*^–/–^ MEFs. As shown, the normalized NLuc-human STING/FLuc ratio decreased gradually and reduced by 75% (8 hours after ds DNA stimulation) and by 90% (12 hours after ds DNA stimulation) (Fig. 4a). The stimulation-dependent production of type I interferons (Fig. 4b) showed that the NLuc-human STING was functional.

Human STING exhibits substantial genetic polymorphism, with multiple variants reported (Yi *et al.*, 2013) (Fig. 4c). To examine the activities of the human variants, we focused on two common variants (HAQ and R232H). The allele frequency of HAQ or R232H is estimated to be 20.4% or 13.7%, respectively. FLuc-P2A-NLuc-human STING (HAQ) or FLuc-P2A-NLuc-human STING (R232H) was stably expressed in *Sting*^–/–^ iMEF and the cells were then stimulated with dsDNA. NLuc-STING (R232H) did not activate TBK1, while NLuc-STING (HAQ) activated TBK1 to some extent (Fig. 4d). Consistently, NLuc-STING (R232H) did not induce the transcription of Cxcl10, while NLuc-STING (HAQ) could induce it to some extent (Fig. 4e). These results suggested that STING (R232H) and STING (HAQ) had lower responsiveness to cyclic dinucleotides (CDNs) that cGAS generates upon activation of dsDNA. Of note, human STING (R232H) was reported to have lower affinity to several CDNs including 2',3'-cGAMP (Vavřina *et al.*, 2021). The normalized NLuc-human STING (R232H or HAQ)/FLuc ratio did not decrease after dsDNA stimulation (Fig. 4f), suggesting virtually no degradation of STING (R232H) and STING (HAQ).

## Discussion

In the present study, we report a quantitative method to monitor STING degradation with two luciferases. The method can be performed on a 96-well/384-well plate, enabling high-throughput screening. The method is not affected by post-translational modifications of STING including phosphorylation and poly-ubiquitination. Therefore, the method has several advantages over western blotting and FACS analysis presently used to monitor STING degradation. Activation of STING can induce cell death with a varying degree of severity in various cell types (Gaidt *et al.*, 2017; Liu *et al.*, 2023; Xun *et al.*, 2024). Having FLuc as an internal control helped quantify STING degradation in live cells accurately. Given that STING undergoes degradation in various cell types including macrophage (Balka *et al.*, 2020; McCauley *et al.*, 2020), FLuc-P2A-NLuc-STING can also be useful to monitor STING degradation in these cells. Human cells, instead of mouse cells, will be ideal to monitor degradation of human STING.

We have recently shown that (i) STING was degraded by ESCRT-dependent lysosomal microautophagy, (ii) STING underwent K63-linked polyubiquitination at REs and this modification was essential for STING degradation, and (iii) STING was loaded into clathrin-coated vesicles from REs, which was the direct substrate of lysosomal microautophagy (Kuchitsu *et al.*, 2023). Despite these advances, there are many questions that remain unsolved regarding a process that regulates STING degradation: What are the trafficking regulators of STING from the Golgi to REs? What is the E3 ligase that K63-polyubiquitinates STING at REs? What regulates the clustering of STING-positive clathrin-coated vesicles? How can lysosomes recognize K63-linked polyubiquitinated STING through the function of ESCRT? A genome-wide screening with siRNAs using the method reported in the present study will answer these questions. A comprehensive insight into the molecular mechanism that regulates STING degradation will also help understand a variety of autoinflammatory and neurodegenerative diseases (Motwani *et al.*, 2019) and may lead to new treatments for these diseases.

Cancer immunotherapy has transformed the treatment of cancer, with a success of immune checkpoint inhibitors of programmed cell death 1 and its ligand (Chamoto *et al.*, 2023). The search for other immune regulators beyond those has been extended to innate immune activation, which is expected to enhance tumour immunogenicity. The cGAS-STING pathway, in particular, in dendritic cells (DCs), has emerged as a critical intrinsic tumour-detecting mechanism (Barber, 2015; Klarquist *et al.*, 2014; Woo *et al.*, 2014). A variety of small chemicals including CDN derivatives (Tian *et al.*, 2024) and non-CDN types (Pan *et al.*, 2020; Ramanjulu *et al.*, 2018) have been developed, and they showed a significant anti-tumour activity in mice. A timely activation and inactivation of STING in DCs will be crucial to proper tumour antigen presentation and stimulation of CD8 T cells. The method reported in the present study may also be beneficial for the screening of chemicals that properly activate human STING.

## Methods

### Antibodies

Antibodies used in the present study were as follows: rabbit anti-phospho-mouse STING (D8F4W; Cell Signaling Technology), rabbit anti-phospho-human STING (E9A9K; Cell Signaling Technology), rabbit anti-phospho-TBK1 (D52C2; Cell Signaling Technology), rabbit anti-TBK1 (ab40676; Abcam), mouse anti-α-tubulin (017-25031; Fujifilm-Wako), Goat Anti-Rabbit IgG (H + L) Mouse/Human ads-HRP (4050-05; Southern Biotech), Goat Anti-Mouse IgG (H + L) Human ads-HRP (1031-05; Southern Biotech), mouse anti-GM130 (610823; BD Biosciences), rabbit anti-STING (19851-1-AP; proteintech), rabbit anti-K63-linked ubiquitin (05-1308; Merck Millipore), Alexa-conjugated secondary antibodies (A21202, A21206, A21207, A21208, A10037; Thermo Fisher Scientific), rabbit anti-clathrin heavy chain (D3C6; Cell Signaling Technology), rat anti-LAMP1 (14-1071-82; Invitrogen), rabbit anti- calreticulin (612137; BD Biosciences), and mouse anti-LgBiT (N710A; Promega).

### Reagents

The following reagents were purchased from the manufacturers as noted: DMXAA (14617, Cayman), MLN7243 (30108, Cayman), and HT-DNA (D6898, Sigma). The plasmids encoding NLuc and FLuc were purchased from Promega.

### Cell culture

MEFs were obtained from embryos of WT or *Sting*^–/–^ mice at E13.5 and immortalized with SV40 Large T antigen. iMEFs were cultured in DMEM supplemented with 10% fetal bovine serum (FBS) and penicillin/streptomycin/glutamine (PSG) in a 5% CO_2_ incubator. iMEFs stably expressing the tagged proteins were established using retroviruses. Plat-E cells were transfected with pMXs vectors and the medium containing the retrovirus was collected. iMEFs were incubated with the medium and then selected with puromycin (2 μg ml^–1^) for several days. RAW-Lucia ISG-KO-STING Cells (InvivoGen) were cultured in DMEM supplemented with 10% FBS, normocin (100 μg ml^–1^), and PS.

### Immunocytochemistry

Cells were seeded on coverslips (13 mm No.1 S, MATSUNAMI), fixed with 4% paraformaldehyde (PFA) in PBS at room temperature for 15 min, and permeabilized with digitonin (50 μg ml^–1^) in PBS or 0.1% TritonX-100 in PBS at room temperature for 5 min. After blocking with 3% BSA in PBS, cells were incubated with primary antibodies followed by secondary antibodies at room temperature for 45 min. When necessary, cells were stained with DAPI. Cells were then mounted with ProLong Glass Antifade Mountant (P36984, Thermo Fisher Scientific).

### Confocal microscopy

Confocal microscopy was performed using LSM880 with Airyscan (Zeiss) with 20 × (0.8NA) Plan-Apochromat dry lens, 63 × (1.4NA) Plan-Apochromat oil immersion lens. Images were analyzed and processed with Zeiss ZEN 2.3 SP1 FP3 (black, 64 bit) (version 14.0.21.201) and Fiji (version 2.9.0/1.54 h).

### Nano-Glo^®^ Dual-Luciferase^®^ Reporter assay

Cells in culture medium were lysed using ONE-Glo^TM^ EX Reagent, in a volume equal to the culture medium, at room temperature. After the measurement of the luminescence of FLuc, an equal volume of NanoDLR^TM^ Stop & Glo^®^ Reagent was added to quench the FLuc signal, and the luminescence of NLuc was measured with GloMax Navigator Microplate Luminometer (Promega, version 3.1.0). The luminescence value of NLuc was divided by that of FLuc to obtain the NLuc/FLuc ratio. The NLuc/FLuc ratios obtained from cells stimulated with DMXAA and the corresponding inhibitor or siRNA were normalized to the averaged NLuc/FLuc ratio obtained from unstimulated cells treated with the corresponding inhibitor or siRNA.

### Type I interferon bioassay

Cell culture supernatants of iMEFs stimulated with DMXAA or HT-DNA were added to Raw264.7- Lucia ISG-KO-STING Cells (Invivogen). Ten hours after incubation, the luciferase activity was measured by GloMax Navigator Microplate Luminometer (Promega, version 3.1.0).

### qRT-PCR

Total RNA was extracted from cells using SuperPrep II (TOYOBO), and reverse-transcribed using ReverTraAce qPCR RT Master Mix with gDNA Remover (TOYOBO). Quantitative real-time PCR (qRT-PCR) was performed using KOD SYBR qPCR (TOYOBO) and LightCycler 96 (Roche). Target gene expression was normalized to the expression of Gapdh.

### Western blotting

Proteins were separated in polyacrylamide gel and then transferred to polyvinylidene difluoride membranes (Millipore). These membranes were incubated with primary antibodies, followed by secondary antibodies conjugated to peroxidase. The proteins were visualized by enhanced chemiluminescence using Fusion SOLO.7S.EDGE (Vilber-Lourmat).

### RNA interference

siRNAs used in the present study were described in the previous study (Kuchitsu *et al.*, 2023). Cells were transfected with siRNAs (5 nM or 20 nM) using Lipofectamine RNAiMAX (Invitrogen) according to the manufacturer’s instructions.

### Statistics and reproducibility

Error bars displayed in bar plots throughout the present study represent the standard error of the mean (SEM) unless otherwise indicated. The SEM was calculated from at least triplicate samples. In box-and-whisker plots, the box bounds the interquartile range divided by the median, one-way analysis of variance followed by Tukey-Kramer post hoc test for multiple comparisons with R (version 4.1.2) and KNIME (version 4.5.1).

### Data availability

The data sets generated and/or analyzed during the present study are available from the corresponding author on reasonable request.

## Author Contributions

T.S., K.S., and Y.K. designed and performed the experiments, analyzed the data, interpreted the results, and wrote the paper; A.S., S.K., R.S., and K.H. performed the experiments; K.M. designed the experiments; T.T. designed the experiments, interpreted the results, and wrote the paper.

## Conflict of Interest

The authors declare no competing financial interests.
